# Pathogenesis of Alcoholic Fatty Liver a Narrative Review

**DOI:** 10.3390/life13081662

**Published:** 2023-07-30

**Authors:** Helmut K. Seitz, Bernardo Moreira, Manuela G. Neuman

**Affiliations:** 1 Centre of Liver and Alcohol Associated Diseases, Ethianum Clinic, Faculty of Medicine, University of Heidelberg, 69120 Heidelberg, Germany; bernardomaikita@gmail.com; 2 In Vitro Drug Safety and Biotechnology, Department of Pharmacology and Toxicology, Temerity Faculty of Medicine, University of Toronto, Banting Institute, Toronto, ON M5G 1L5, Canada; m_neuman@rogers.com

**Keywords:** alcoholic fatty liver, adenosine monophosphate activated kinase, sterol regulatory element binding protein 1c, peroxisome proliferator activated receptor α, oxidative stress, cytochrome P450 2E1

## Abstract

Alcohol effect hepatic lipid metabolism through various mechanisms, leading synergistically to an accumulation of fatty acids (FA) and triglycerides. Obesity, as well as dietary fat (saturated fatty acids (FA) versus poly-unsaturated fatty acids (PUFA)) may modulate the hepatic fat. Alcohol inhibits adenosine monophosphate activated kinase (AMPK). AMPK activates peroxisome proliferator activated receptor a (PPARα) and leads to a decreased activation of sterol regulatory element binding protein 1c (SRABP1c). The inhibition of AMPK, and thus of PPARα, results in an inhibition of FA oxidation. This ß-oxidation is further reduced due to mitochondrial damage induced through cytochrome P4502E1 (CYP2E1)-driven oxidative stress. Furthermore, the synthesis of FAs is stimulated through an activation of SHREP1. In addition, alcohol consumption leads to a reduced production of adiponectin in adipocytes due to oxidative stress and to an increased mobilization of FAs from adipose tissue and from the gut as chylomicrons. On the other side, the secretion of FAs via very-low-density lipoproteins (VLDL) from the liver is inhibited by alcohol. Alcohol also affects signal pathways such as early growth response 1 (Egr-1) associated with the expression of tumour necrosis factor α (TNF α), and the mammalian target of rapamycin (mTOR) a key regulator of autophagy. Both have influence the pathogenesis of alcoholic fatty liver. Alcohol-induced gut dysbiosis contributes to the severity of ALD by increasing the metabolism of ethanol in the gut and promoting intestinal dysfunction. Moreover, pathogen-associated molecular patterns (PAMPS) via specific Toll-like receptor (TLR) bacterial overgrowth leads to the translocation of bacteria. Endotoxins and toxic ethanol metabolites enter the enterohepatic circulation, reaching the liver and inducing the activation of the nuclear factor kappa-B (NFκB) pathway. Pro-inflammatory cytokines released in the process contribute to inflammation and fibrosis. In addition, cellular apoptosis is inhibited in favour of necrosis.

## 1. Introduction

Alcohols are a group of hydrocarbons containing one or more hydroxyl (-OH) groups. Ethanol (C_2_H_5_OH, ethyl alcohol) is the main psychoactive ingredient in alcoholic beverages. Under usual conditions, beverages produced by fermentation have an alcohol concentration of maximum 14%. By distillation, the fermented mixture is a pure condensate. Alcohol is a sedative with hypnotic effects. Alcohol intoxication may result in amnesic syndrome, long-term dependence, and a variety of physical and mental disorders [1,2]. 

A volume of beverage alcohol (e.g., a glass of wine, a can of beer, or a mixed drink containing distilled spirits) that contains approximately the same amounts (in grams) of ethanol regardless of the type of beverage is termed a “standard drink.” The term is often used to educate alcohol users about the similar effects associated with consuming different alcoholic beverages served in standard-sized glasses or containers (e.g., the effects of one glass of beer are equal to those of one glass of wine). In the United Kingdom, the term “unit” is employed, where one unit of an alcoholic beverage contains approximately 8–9 g of ethanol; in North American literature, “a drink” contains about 12 g of ethanol. In other countries, the amounts of alcohol chosen to approximate a standard drink may be greater or less, depending on local customs and beverage packaging.

### 1.1. Organ-Induced Damage

Alcohol-related mental and behavioural disorders are classified as psychoactive substance use disorders in International Classification of Diseases (ICD-10). Other organ-induced damage is represented by cardiomyopathy, alcoholic hepatitis, pancreatitis, and injury produced to other systems. The physiological health consequences of alcohol consumption are closely correlated with the pattern of drinking. The first pattern of drinking is moderate alcohol consumption (in the USA defined as no more than one drink/day for women, and no more than two drinks/day for men). The second pattern of drinking which could be harmful to health is high-risk drinking. Organ toxicity is mostly associated with the second pattern of drinking [1].

### 1.2. Fatty Liver

Fat accumulation in the liver is an early and frequent response of the liver towards chronic alcohol consumption. Over 90% of regular alcohol consumers over a longer period of time develop fatty liver (FL) [1,2]. In addition, FL also occurs in binge drinkers. These are individuals who consume large amounts of alcohol within hours [2]. This effect has been shown in animal experiments. As soon as 12 h after the application of a high dose of alcohol to mice, a 10-fold increase in hepatic triglycerides has been observed [3].

Histologically, FL is characterized by the deposition of fat droplets in the cytoplasm of hepatocytes. FL is defined as a liver in which more than 5% of hepatocytes reflect fat deposition. Hepatic fat accumulation after alcohol consumption occurs primarily around the central vein, from where it extends to the centre of hepatic lobule and, finally, towards the portal area. In alcoholics, steatosis is predominantly macro-vesicular and rarely micro-vesicular. Chemically, the deposition of triglycerides in fat droplets of hepatocytes is very characteristic [4].

Following alcohol abstinence, alcoholic FL disappears quickly [5,6,7]. Steatosis is not associated with any symptoms. This is why alcoholic FL has been classified as a side effect of alcohol consumption and as a harmless precondition of alcohol-induced liver damage. However, under certain conditions alcoholic fatty liver may progress to inflammation, fibrosis, and cirrhosis [1]. Data emphasize the fact that free fatty acids (FFAs) play a pathological role in this process [2]. Therefore, it is of major significance to understand the effect of alcohol on fatty acid metabolism and the mechanisms by which alcohol induces FL. 

Under physiological conditions, fatty acids from circulation enter the hepatocytes. The neo-synthesis of fatty acids on one side, as well as the degradation of fatty acids and their secretion on the other side, keep a certain balance. If one of these factors changes, it will be compensated by another mechanism. For example, an increased availability and an increased uptake of fatty acids into the hepatocyte are followed by an increased ß-oxidation to acetyl-CoA which can then be utilized in the citric cycle for energy generation. Acetyl-CoA can also be used for the generation and secretion of ketone bodies to other organs, for example, to muscle cells and the brain, where the ketone bodies are used for energy production. If no energy is required, fatty acids are esterified to triglycerides, stored in fat droplets and then oxidized when needed or secreted in the form of very-low-density lipoproteins (VLDL) into the circulation [8,9,10]. 

**Figure 1:**
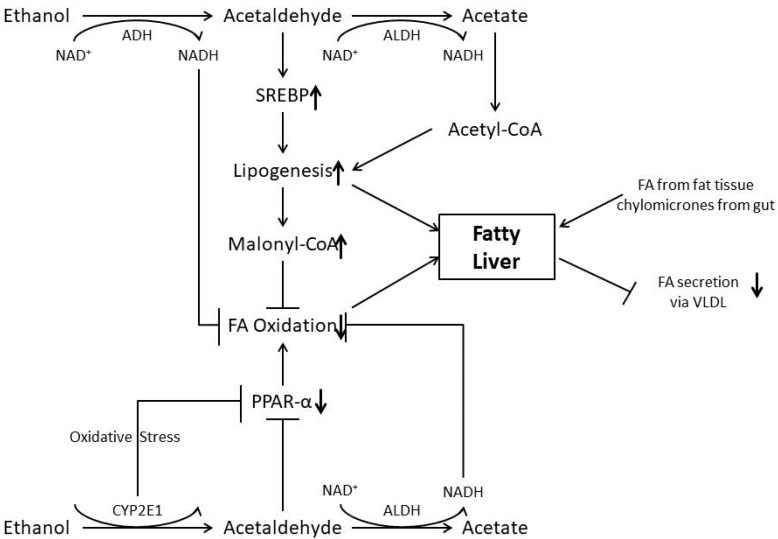
Mechanisms involved in the pathogenesis of alcoholic fatty liver.

Therefore, under physiological conditions, intercellular lipid concentrations are observed only for a short period of time. Alcohol, however, disturbs the homeostasis of lipid metabolism by various mechanisms and, as a consequence, fat is accumulated in the liver (Figure 1). 

Alcohol (Ethanol) is metabolized in the liver by alcohol dehydrogenase in acetate. Acetate dehydrogenase further metabolize this product in acetate, which is a toxic product. Cytochrome P450 2E1 contributes to ethanol oxidation leading to lipogenesis.

This review describes metabolic and molecular factors inducing alcoholic FL. For more details, the reader is referred to the following review articles [1,2,8,9,10,11,12].

### 1.3. Types of Alcoholic Drinks

The type of alcoholic drink consumed impacts liver damage by adding new converges. There is an association between the drink, its specific taste, scent and these congeners. The specific taste of the drink has an influence on the consumer preference and consumption of alcohol. An alcohol congener’s interaction with the human organism follows different pathways in comparison to ethanol concentration [10]. Product flavour is an important component in product selection, primarily endorsing preference for sweet e-liquid flavour (e.g., fruit) in women and young adolescents, compared with preferences reported by older adults [10]

## 2. Metabolism of Ethanol and Ethanol as a Source of Energy

The physiological burning value of pure alcohol is 30 kJ/g, which is approximately 7 kcal/g ethanol. This means for example that 500 mL beer with a 5% volume of alcohol content has approximately 140 kcal. Furthermore, each beverage also has, in addition to its ethanol content, calories from other components such as sugar as a source of energy [13].

### 2.1. Ethanol Metabolism

Ethanol cannot be stored in the body. Ethanol is primarily (over 90%) metabolized in the liver and is oxidized via alcohol dehydrogenase (ADH) and aldehyde dehydrogenase (ALDH). These enzymes are found in the cytoplasm of hepatocytes. Chronic alcohol consumption induces cytochrome P450 2E1 in the endoplasmic reticulum of the hepatocytes. This so called microsomal ethanol oxidizing system (MEOS) also generates acetaldehyde (AA) [14]. Only a small percentage of ethanol is oxidized through catalase in the peroxisomes of the hepatocyte. 

Regardless of the metabolic pathway, AA is further metabolized to acetate in the mitochondria by aldehyde dehydrogenases (ALDH). 

After binding to coenzyme A, acetate is further synthesized to acetyl-CoA through acetyl Co-A-synthetase. Both ADH and ALDH need nicotinamide-adenine dinucleotide (NAD^+^) as a co-enzyme, which is then transferred into its reduced form nicotinamide-adenine dinucleotide-hydrogen (NADH). Thus, the oxidation of ethanol leads to an excess of NADH and acetyl Co-A [1,15]. 

### 2.2. Acetyl Co-A 

Acetyl Co-A can be generated from carbohydrates such as glucose, but not the other way around. In an excess of acetyl Co-A, it is used either to generate energy in the citric cycle, or for the synthesis of fatty acids. NADH, however, which also exists in excess, inhibits the citric cycle. This is the reason why acetyl Co-A from ethanol oxidation is predominantly used for the synthesis of fatty acids (FAs). FAs are esterified with glycerol to triglycerides and stored as fat drops in the cytosol of the hepatocyte [1,15].

Steatosis is also promoted by excess dietary lipids and can be attenuated by their replacement with medium-chain triglycerides [16]

## 3. Inhibition of Fatty Acid Oxidation

Due to the fact that ethanol metabolism produces NADH and decreases the NAD^+^/NADH ratio, other metabolic pathways leading to hepatic steatosis are activated [15]. The result is a change in the cellular redox potential with an inhibition of the oxidative degradation of fat in the hepatocytes, since many enzymes of fat acid oxidation require NAD^+^ and are inhibited by higher NADH levels [10].

### 3.1. Redox

For a long time, the redox change in NAD^+^/NADH ratios was seen as a major cause for FL. Such a metabolic change, however, may not explain completely the fast increase in hepatic fat by acute alcohol consumption. In addition, experimental data show that the change in the NAD^+^/NADH ratio cannot explain hepatic fatty liver induced by alcohol alone since animal studies have shown that ethanol may lead to hepatic fat deposits even after normalization of the NAD^+^/NADH ratio [17].

The pathogenesis of alcoholic FL is a multifactorial process, requiring in addition to metabolic changes various signal transduction pathways which are also changed and which affect not only the degradation of fat and fatty acids, but also other components of lipid metabolism. Alcohol leads to a depolarization of mitochondria. The consequences of mitochondrial depolarization leads to reduced energy generated from adenosine triphosphate (ATP). In addition, this depolarization leads to a decreased influx of fatty acids into the mitochondria with a consecutively reduced ß-oxidation [18].

### 3.2. Transcriptional Factors

Alcohol has an effect on the transcriptional factor peroxisome proliferation-activated receptor α (PPAR α) [19]. PPAR α is a nuclear receptor interacting with the retinoid × receptor. The protein complex is activated through a binding of fatty acids and leads to an increased expression of genes responsible for the transport and oxidation of fatty acids. Animal experiments and cell cultures have shown that alcohol application leads to a decreased binding of PPAR α to promotor regions and, consecutively, to a decreased expression of coded protein. 

Acetaldehyde deactivates PPAR α, possibly through a covalent binding or the production of protein adducts which inhibit the binding to the promotor elements [19]. Additionally, alcohol directly deactivates PPAR α through CYP2E1-mediated oxidative stress. Other factors which inhibit PPAR α are NADH/NAD or via the inhibition of adiponectin, AMPK, and zinc levels. The importance of the inhibitory effect of alcohol on PPAR α for the generation of FL has been shown in animal experiments, where the application of PPAR α agonists inhibits alcohol-mediated FL [20].

## 4. Enhancement of De Novo Lipogenesis

Acute and chronic alcohol consumption increases the synthesis of fatty acid in the hepatocytes. This alcohol effect results from an increased expression of enzymes involved in lipogenesis, such as fatty acid synthase (FASN), acyl Co-A carboxylase (ACC) and ATP citrate lyase (ACL), or stearyl-CoA desaturase. These enzymes are coded through genes which are regulated through the transcription factor sterol-regulatory element binding protein-1 (SREBP-1) [21]. Alcohol induces in tissue cultures, in vitro, and in the animal model an increased SREBP-1 level [21]. Serine-arginine-rich protein kinase 2 (SRPK2), a key kinase controlling alternative splicing, is activated in hepatocytes in response to alcohol, in mice with chronic-plus-binge alcohol feeding, and in patients with ALD. Such induction activates sterol regulatory element binding transcription factor 1 (SREBP-1) and promotes lipogenesis in ALD. 

### 4.1. Growth Factors

Fibroblast growth factor 21 (FGF21) is upregulated in alcohol induction of SRPK2. This results in steatosis, lipotoxicity, and inflammation. Mechanistically, SRPK2 is required for alcohol-mediated impairment of serine-arginine splicing factor 10 (SRSF10), which generates exon 7 inclusion in lipin 1, and triggers the concurrent induction of lipogenic regulators—lipin 1β and SREBP-1. FGF21 suppresses alcohol-induced SRPK2 accumulation through mTORC1 inhibition-dependent degradation of SRPK2 [10,21].

### 4.2. Coenzymes

The effect of alcohol is mediated through an alcohol-mediated inhibition of AMPK or primarily by acetaldehyde [19,22]. In this context, it is important to note that increased concentrations of malonyl CoA are produced. malonyl CoA allosterically inhibits carnitin-palmitoyl-transferase 1 (CPT-1). CPT-1 is a key factor in the uptake of fatty acids and fatty acid-Acyl-CoA from the plasma into the mitochondria. Thus, the oxidation of fatty acids is inhibited allosterically through alcohol-induced lipogenesis and malonyl-CoA, which is a further mechanism by which alcohol affects fat degradation in the liver negatively.

In addition to an inhibition of AMPK by acetaldehyde, SREBP1c is also activated by lipopolysaccharides, 2-arachidonoylglycerol (2-AG), complement, endoplasmic reticulum stress, reduced adiponectin, NAD-dependent protein deacetylase sirtuin 1 (SIRT1), signal transducers and activator of transcriptor 3 (STAT3), and H3K9 (trimethylation of lysine 9 of histone 3) methylation [2,21,22]. 

## 5. The Role of Cytochrome P4502E1

It has been shown in cell cultures of human hepatocytes that the induction of CYP2E1 correlated significantly with the degree of hepatic steatosis [23]. Furthermore, hepatic fat content was found to be significantly reduced in CYP2E1 knockout mice following alcohol administration when compared to wild-type animals [24,25,26].

### P4502E1

Cytochrome P4502E1 may play an important role in the pathogenesis of alcoholic fatty liver [27,28,29]. The NADPH oxidase activity of CYP2E1 can stimulate NADPH transport into the mitochondria. In addition, ROS generated by CYP2E1 may damage mitochondria leading to an inhibition of ß-oxidation [28]. Other mechanisms of CYP2E1-mediated hepatic steatosis may include an impairment of protein kinase B activity by CYP2E1—mediated oxidative stress [29]—and a suppression of PPAR a by oxidative stress [25]. The non-competitive CYP2E1 inhibitor, clomethiazole, attenuates acute ethanol-induced hepatic steatosis by suppressing oxidative stress, an adiponectin decline, and an activation of autophagy [30]. In patients with alcoholic fatty liver, clomethiazole administered for 1 week improved hepatic steatosis only [31]. 

## 6. Cytochrome P4502A5 and Nicotine

Cytochrome P4502A5 (CYP2A5) is also induced by ethanol and this induction is regulated by nuclear factor-erythroid 2-related factor 2 (NRF2) [32]. CYP2A5 knockout mice develop more severe hepatic steatosis than wild-type animals [32]. A complex cascade through PPARa is responsible for this effect. Interestingly, alcohol and tobacco are often misused together. Recent data have shown that alcoholic fatty liver was enhanced by nicotine in cyp2a5+ mice, but not knockout mice. Nicotine is metabolized through CYP2A5, and both ethanol and nicotine induce CYP2A5. The mechanisms by which nicotine enhances alcoholic fatty liver are mediated through CYP2A5-induction-enhanced nicotine metabolism and ROS generation [33].

## 7. Effect of Alcohol on Fat Tissue

A further mechanism by which alcohol contributes to fatty liver is its effect on fat tissue. Fat tissue is an important source of energy. Food calories are stored as fat in adipocytes. In the fasting state or under exercise conditions, adipocytes liberate the stored fat. Animal data show that chronic alcohol consumption increases lipolysis in fat tissue. An increased degradation of triglycerides to free fatty acids, which are secreted into the circulation. As a consequence, the mass of body fat decreases. On the other hand, fatty acids secreted from fat tissue are taken up by the liver and esterified with glycerol to triglycerides. Thus, a shift of triglycerides in adipocytes to triglycerides in hepatocytes occurs, which increases hepatic fat deposition in the liver [34]. 

It is interesting to note that alcoholics with fatty liver have a significantly lower body weight compared to control persons. They also have a lower body mass index (BMI) and lower body fat. Alcohol consumption results in a decreased production of adiponectin, which is generated by adipocytes. Ethanol-induced oxidative stress *via* CYP2E1 disrupts adiponectin secretion from adipocytes [35]. Adiponectin is secreted from adipocytes into the circulation and its serum concentration is significantly reduced in heavy drinkers. Carnitine palmitoyl transferase 1 (CPT1) is the enzyme responsible for fatty acid oxidation, as the key target of adipokines. The physiological effect of adiponectin includes an increase in CPT1 activity, as well as the oxidation of fatty acids, while the activity of acetyl CoA carboxylase and fatty acid synthetase, two key enzymes of de novo lipogenesis, is inhibited [36,37,38]. In animal experiments, alcohol-induced hepatic FL could be significantly inhibited by the administration of recombinant adiponectin [38].

## 8. Disturbed Lipid Transport 

Stored lipids or triglycerides can be exported into the circulation from the hepatocytes in the form of very low-density lipoproteins (VLDL) only. Before secretion, stored triglycerides in the fat droplets are split and free fatty acids (FFA) are generated. These fatty acids are then loaded with Apolipoprotein B100 in the endoplasmic reticulum together with phospholipids and cholesterol and finally synthesized to VLDL particles [39]. 

PPAR α regulates not only genes involved in fat oxidation, but also regulates the expression of key factors in fat transport such as Apolipoprotein B, triglyceride transfer protein, and fatty acid binding protein (FABP). By inhibiting PPAR activity, alcohol leads to disturbances of VLDL generation and secretion with the intercellular accumulation of fatty acids as a consequence [40].

## 9. Effect of Alcohol on Various Intracellular Signal Pathways

### 9.1. AMP Activated Protein Kinase (AMPK)

Alcohol inhibits AMP-activated protein kinase (AMPK) [17]. AMPK is an enzyme that protects cells against ATP deficiency, or energy deficiency. AMPK is regulated through AMP and ATP concentrations in the cells. AMP is a degradation product of ATP and therefore an excellent indicator for energy deficiency. AMPK regulates energy deficiency through a shutdown of energy-effective biosynthesis. For example, AMPK inhibits enzymes responsible for cholesterol and fatty acid synthesis through phosphorylation, such as Acetyl CoA carboxylase (ACC). ACC is a key enzyme of lipid synthesis. ACC catalyses the carboxylation of acetyl CoA to malonyl CoA, the initial product for de novo lipogenesis. At the same time, malonyl CoA inhibits fat burning allosterically. Additionally, the oxidation of fatty acids is stimulated through a diminished malonyl CoA concentration due to increased AMPK activity. This process as well as further ATP-generating catabolic processes, such as the citric cycle or glycolysis, are activated directly through AMPK as a counter regulation in the presence of energy deficiency. Fat burning is inhibited and lipogenesis stimulated through the inhibitory effect of alcohol on AMPK, independent of the real energy need of the cell. This effect of alcohol is due to the stimulation of SREBP1-levels. Alcohol-induced hepatic fat accumulation can be significantly inhibited through the application of an AMPK-activator such as metformin [22].

### 9.2. Early Growth Response 1 (Egr-1)

Alcohol affects hepatic lipid accumulation through a further transcription factor, namely early growth response 1 (Egr-1) [41]. Egr-1 regulates the expression of genes, which are induced by cellular stress (alcohol causes cellular stress by various mechanisms). Egr-1 increases the expression of tumour necrosis factor α (TNFα). TNF-α is lipogenic, through activation of SHREP1, which leads to an increased de novo lipogenesis. 

Alcohol also activates Kupffer cells to release pro-inflammatory cytokines such as TNF-α that promote fat accumulation in the liver. Another activation of Kupffer cells may occur through pathogen-associated molecular patterns (PAMPs) from alcohol-induced gut bacterial overgrowth and dysbiosis. The importance of Egr-1 for alcohol-induced fatty liver could be clearly shown in animal experiments. Egr-1-deficient mice do not show an increase in TNF-α expression and hepatic fat accumulation is prevented after alcohol administration [41]. 

## 10. The Mammalian Target of Rapamycin (mTOR) of Autophagy

Autophagy is a genetically determined evolutionarily conserved process by which cells degrade their own cellular constituents. Lipophagy is a special form of autophagy and plays an important role in the initiation of fat burning. During lipophagy, the fat droplets have to be cracked and triglycerides have to be liberated. For this process, fat droplets are included in autophagosomes. Autophagosomes have a double membrane and transport their load to liposomes and merge with them. Fat is digested through liposomal enzymes. The fatty acids can be oxidized via ß-oxidation in the mitochondria. The alcohol affects autophagy [42,43]

The mTOR signal pathway is a key regulator of autophagy. Studies have shown different effects of alcohol on mTOR activation and autophagy. The long-term application of alcohol inhibits autophagy, while short-term application results in an increased autophagy. During acute alcohol exposition or in an acute phase of alcoholic liver damage, the inhibitory effect of hepatic steatosis is induced. On the other hand, the alcohol effect of ß-oxidation is inhibited. Thus, an accumulation of toxic free FFAs in the cytoplasm of hepatocytes could be induced through the induction of lipophagy [34,40,44].

## 11. Endotoxin and Presepsin 

Chronic alcohol consumption damages almost all tissues in the human body, which may additionally enhance fatty liver, for example effecting the glucose metabolism, the endocrine pancreas, or the intestinal barrier function. Intestinal CYP2E1 may be a mediator of alcohol-induced intestinal leakiness [45] with the uptake of endotoxins resulting in fatty liver. The gut microbiota, which is changed by chronic alcohol consumption, may also play an important role in hepatic steatosis and alcoholic liver disease [46,47]. 

### 11.1. Alcohol and Endotoxin

ALD is associated with elevated plasma endotoxin levels in alcoholic liver [48,49,50]. The endotoxemia results in an increase level of lipopolysaccharides (LPS) [51]. Endotoxemia increases intestinal permeability [52]. 

Ethanol effects glycosylation of epithelial mucins, which alters the protective mucus layer and may cause a change in adherent bacterial species [53]. The effect of alcohol on intestinal permeability is in part due to the bacterial metabolism of ethanol to acetaldehyde [53]. 

Patients with alcoholic liver disease have an overgrowth of *Candida* sp. compared to non-alcoholic controls [54,55,56,57,58]. Moreover, 2 weeks of abstinence from alcohol reduces the proportion of *Candida albicans* in patients with alcohol-use disease. In addition, mice fed a chronic diet supplemented with alcohol have an increase in *Meyerozyma guillermondii* [59].

### 11.2. Alcohol and Presepsin

A very useful biomarker in monitoring LPS is presepsin. The presepsin molecule is characterized by rapid kinetics: its activation time is only 2 h following a bacterial or fungal event, with a peak concentration after 3 h. Presepsin and resistin are better markers for bacterial infection in patients with decompensated liver cirrhosis [60]. 

The presepsin secretion sCD14-ST is a 13 k Da fragment derived from cleavage of CD14, a glycoprotein of 55 k Dalton anchored to the membrane of monocytes, macrophages, and polymorphic neutrophils. CD14 acts as a receptor for LPS complexes and the specific LPS binding protein (LBP). It can bind to peptidoglycans and other surface structures present in both Gram-positive and Gram-negative bacteria. Once bound to the LPS-LBP complex, it activates the intracellular inflammatory response of the TLR4/MD2-complex, triggering the host’s inflammatory cascade against the infectious pathogenic agent. Phagocytosis and activity of plasma proteases (lysosomal enzymes, cathepsin D) result in the formation of the fragments CD14 subtype, in particular the 13 kDa fragment of sCD14-ST [60].

## 12. The Intestinal Microbiome 

The intestinal microbiome is now recognized as playing a central role in the proper functioning of the intestine. The GI microbiome consists of bacteria, fungi, and viruses. The major portion of the microbiome is located in the ileum and proximal colon. The multiple effects of the gut microbiota influence physiological function [61].

Gut microbial imbalance are alterations in the small intestinal flora, changes in the ratio of useful to harmful bacteria and the translocation of colonic bacteria producing dysbiosis. The dysbiosis increases intestinal permeability to endotoxin and has a key role in the metabolic changes leading to pathogenesis. In the experimental mouse model, the increase endotoxin levels resulted in the decreased production of long-chain fatty acids in being downregulated in the liver along with let-7a and let-7b. The treatment of human hepatic stellate cells (HSCs) with LPS and TGF-β similarly decreased the expressions of let-7a and let-7b. Conversely, the overexpression of let-7a and let-7b suppressed the myofibroblastic activation of cultured human HSCs induced by LPS and TGF-β, as evidenced by repressed ACTA2 (α-actin 2), COL1A1 (collagen 1A1), TIMP1 (TIMP metallopeptidase inhibitor 1), and FN1 (fibronectin 1) [62].

ALD is associated with elevated endotoxin levels in patients and rodent models of alcohol consumption [62,63,64]. LPS binds to TLR4. The TLR4-deficient mice develop less liver injury following alcohol exposure as compared to wild-type mice, suggesting a role for bacterial metabolism in the pathogenesis of liver injury from alcohol [65]. Increased endotoxemia produces intestinal permeability [66].

Finally, alcohol-induced changes in the microbiome enhance hepatic steatosis. 

Alcohol affects lipid metabolism through various mechanisms, leading synergistically to an accumulation of fatty acids and triglycerides. Overweight and obesity as well as the quality of dietary fat (saturated FA versus PUFA) may modulate the quantity and quality of hepatic fat. Alcohol inhibits adenosine monophosphate activated kinase (AMPK). AMPK activates PPARα and leads to a decreased activation of SRABP1c. Ethanol toxicity is enhanced by the presence of copper or iron, which promote lipid peroxidation. Impairment of mitochondrial function is an early step in the ethanol-induced toxicity.

In Figure 1 we present a schematic of a possible scenario of the mechanism of the alcohol-induced toxic effect on liver cells. 

## 13. Inflammasome

The proinflammatory cytokines are activated by alcohol signalling for inflammation via multi-faceted innate immunity-activating alarmin. Ethanol signals *via* Tall Receptors (TLR2, TLR4, TLR9)**,** and chemokine receptor (CXCR4) to activate inflammation. It also induces the release of interleukines (IL-1α, IL-1β, IL-8, IL-6), TNF-α, interferon (IFN)-γ, as well as chemolines (CXCL11 and CXCL12) [65,66].

Ethanol enhances inflammation in the liver by activating hepatic stellate cells (HSCs). HSCs reside in the perisinusoidal space between sinusoidal endothelial cells and hepatocytes. HSCs and Kupffer cells integrate cytokine-mediated inflammatory responses in the sinusoids [67,68,69]. NF-κβ can also induce the antiapoptotic genes (TRAF-1 and TRAF-2) influencing the appearance and the growth of HCC. Increased TNF-α production has been shown to deregulate the tight junction in the GI tract, causing disruption of the intestinal barrier. 

The change induced by acetaldehyde and ROS is related to folate metabolism. ALD patients have been found with polymorphisms in the methylene tetrahydrofolate reductase gene, leading to an alteration in folate metabolism and HCC development. Ethanol effects the glycosylation of epithelial mucins, which may cause a change in adherent bacterial species [70]. Germ-free mice studies suggest that the effect of alcohol on intestinal permeability is in part due to the metabolism of ethanol to acetaldehyde [71]. Other bacterial metabolites also effect gut-barrier disruption [72]. The changes in the bacterial variety of the host induced by ALD brings with it an important modification in the composition of bile acids and their concentration. The bacterial dehydroxylation results in an increase in Deoxycholic Acid synthesis. Accumulation of bile acids and the activation of the farnesoid X receptor (FXR) induce the expression of the bile-salt-export pump and organic solute transporter alpha and beta. 

Alcohol administration to rats resulted in a decrease in the *Lactobacillus* sp., *Pediococcus* sp., *Leuconostoc* sp., and *Lactococcus* sp. [73]. Alcohol-fed mice also developed an increase in *Proteobacteria* sp. and *Actinobacteria* sp. [74]. Alcoholic individuals present a decrease in the abundance of Bacteroidetes together with an increase in Enterobacteriaceae and Proteobacteria [58] as well as decreased fungal species richness [75,76,77]. Two weeks of abstinence from alcohol reduces the proportion of *Candida albicans* in patients with alcohol-use disease [78]. 

The examination of the stools from alcoholic patients has shown a correlation between the levels of long-chain fatty acids (LCFA) and the numbers of Lactobacilli in the stools. The Lactobacilli in the intestinal microbiome (IM) metabolize saturated LCFAs, which promote the growth of the bacteria. Dietary fat composition influences the IM and modulates liver injury. In a murine model, the administration of a diet rich in unsaturated fats resulted in a decrease in Bacteroidetes and an increase in Proteobacteria and Actinobacteria, resulting in a more severe ALD. In addition, there were metabolomic changes—a decrease in long-chain fatty acids (LCFA)s, medium-chain fatty acids (MCFA)s, and short-chain fatty acids (SCFA)s. the supplementation of saturated LCFAs maintains intestinal eubiosis and reduces ethanol-induced liver injury in mice [79]. Thus, dietary lipids have a role in dysbiosis, leading to the development of ALD, enhancing the severity of the disease, or reducing inflammation [80,81,82,83,84,85,86]. 

## 14. The Significance of Alcohol Consumption to Liver Toxicity

The role of alcohol in the aetiology of liver disease has seen the establishment of several codes for categories of liver diseases primarily caused by alcohol. For example, ICD-10(7) recognizes several forms of alcoholic liver disease (ICD-10, K70), sometimes considered stages that range from reversible alcoholic steatosis (K70.0) and alcoholic hepatitis (K70.1), to alcoholic fibrosis and sclerosis of the liver (K70.2), and further to severe and irreversible stages such as alcoholic liver cirrhosis (K70.3) and alcoholic hepatic failure (K70.4). Alcohol consumption, in particular heavy use over time, has been central in the aetiology and development of these diseases [87,88,89]. However, liver diseases are multifactorial, and alcohol use may play a role in the progression of all types of cirrhosis [90]. Even one drink per day may have an effect on the incidence of liver cirrhosis [91]. In the scientific review of liver-related diseases, including cirrhosis, it is important to include both alcohol- and non-alcohol-induced liver injury when examining the impact of alcohol use [92,93,94,95,96,97]. 

In the meta-analysis performed by us, alcohol consumption showed a complex association with hepatic steatosis, with substantial differences according to ethnicity and sex. Low alcohol consumption was beneficial in Japan with good epidemiological evidence. There was no association in other countries. However, heterogeneity was large in countries other than Japan [94,95].

Several groups demonstrated a link between alcohol consumption and mortality among women drinking high amount of alcohol [96,97,98,99]. 

Becker U. et al. explained the lower risk for alcohol-induced cirrhosis in wine drinkers [100]. The authors prospectively studied 30,630 participants from the Copenhagen area. The following parameters were noted: weekly intake of beer, wine, and spirits. The participants’ data (sex, age, body mass index, smoking habits, and education) were noted. The first hospital admission or death due to alcohol-induced cirrhosis were obtained from the National Hospital Discharge Register. The individuals who drank more than 5 drinks per day had a higher risk for developing cirrhosis compared with non- or light drinkers. Additionally, individuals who drank no wine and individuals drinking 16% to 30% wine out of their alcohol total intake had a higher risk of developing cirrhosis compared to the people drinking 51% or more of wine. The authors suggest that wine drinkers are at a lower risk than beer and spirits drinkers [100].

### Biological Mechanisms That Contributed to Alcohol-Related Research 

Many scientists have had a specific interest in understanding the role of polyphenols in wine, especially flavonoids, which stimulated research into their potential benefits to human health. One of the main properties of polyphenols is their antioxidant activity, which enables them to attenuate the development of atherosclerosis, inflammatory diseases, and cancer. Wine contains trans-resveratrol, which may be the most effective anticancer polyphenol present in red wine as consumed by healthy human subjects [101]. A group of clinical chemists tested the absorptive efficiency of three of these constituents (trans-resveratrol, [+]-catechin, and quercetin) when given orally to healthy human subjects in three different media. The absorption of these polyphenols was equivalent in aqueous and alcoholic matrices, but at peak concentrations was inadequate to permit circulating concentrations consistent with biologic activity. The author concluded that the literature reporting powerful in vitro anticancer and anti-inflammatory effects of the free polyphenols is irrelevant, given that the polyphenols are absorbed as conjugates [102]. Moreover, specific methods to determine the possible interactions of white and red wines with cork have been established in a biochemical laboratory [103]. The importance of poly-phenols was also studied in people with different medical condition [104].

However, after 30 years of dedicated research there is a lack of pharmacological or human evidence confirming the polyphenols’ biological actions, since in each type of grape the quantity and quality of polyphenols differ. Moreover, the fermentation process differs from one winery to another. Future research will eventually clarify the positive activities of the compounds. There is a clear recommendation to responsibly drink moderate amounts of wine with meals.

## 15. Future Research

The liver has a unique blood supply from both portal and systemic sources. Portal blood is delivered to the liver directly via absorption from the GI tract. The future research will include both substances that are ingested and also substances that are either produced or metabolized by the intestinal microbiome.

Hepatic steatosis is a result of either changes in hepatic metabolism linked to insulin resistance, diabetes, metabolic syndrome, or obesity leading to metabolic syndrome. Steatosis may progress via steatohepatitis, fibrosis, and cirrhosis, and its complications include hepatocellular carcinoma.

## 16. Conclusions

The metabolism of alcohol is involved in alcoholic fatty liver disease leading to tissue changes. There are ethnic, sex, age, and environmental differences. Alcohol-induced toxic metabolites interfere with the mitochondrial regulation pathways, which is important in understanding ALD pathogenesis. In addition, alcohol has the capacity to disturb gut microbiota, favouring the expansion of endotoxin-producing bacteria and intestinal permeability, leading to inflammation and fibrogenesis. Different biomarkers are used to define the severity of the disease and its possible repair. The unique liver blood supply, from both portal and systemic sources, includes ingested and substances that are either produced or metabolized by the intestinal microbiome.

Ethanol is oxidized via the CYP 2E1; acetaldehyde in the presence of acetaldehyde dehydrogenase generates NADH and is acetated, which inhibits FA oxidation. Acetaldehyde stimulates SREBP and inhibits PPARα. While increased SREBP stimulates lipogenesis, decreased PPARα inhibits FA oxidation, and so does the addition of malonyl-CoA. Oxidative stress generated by ethanol oxidation through cytochrome P4502E1 also inhibits PPARα and may damage mitochondria with a decrease in NADH and acetaldehyde oxidation. In addition, FA and chylomicrons are delivered from fat tissue and from the gut, respectively. 

Finally, FA secretion as VLDL from the hepatocytes is inhibited by alcohol. NAD (nicotine adenine dinucleotide); NADH (nicotine adenine dinucleotide) in reduced form; ADH (alcohol dehydrogenase); ALDH (acetaldehyde dehydrogenase); FA (fatty acid) SREBP1c; PPARα; CYP2E1 (cytochrome P4502E1); VLDL (very low density lipoprotein).

## Data Availability

Not applicable.

## Abbreviations

2-AG	2-arachidonoyl-glycerol
AA	acetaldehyde
Acetyl	acetyl-Coenzyme A
ACC	CoA acyl Co-A carboxylase
ACE-2	angiotensin-converting enzyme 2
ACL	ATP citrate lyase
ACTA-2	α-actin 2
ADH	alcohol dehydrogenase
ALDH AH	acetaldehyde dehydrogenase alcoholic hepatitis
AIH	autoimmune hepatitis
ALD	alcoholic liver disease
ALI	acute liver injury
ALP	alkaline phosphatase
ALT	alanine aminotransferase (glutamic-pyruvic transaminase, GPT)
AMA	anti-mitochondrial antibody
AMPK	adenosine monophosphate activated kinase
ANA	antinuclear antibody
AST	aspartate aminotransferase (glutamic-oxalic- transaminase, GOT)
ASH	alcoholic steatohepatitis
ATP	adenosine triphosphate
BMI	body mass index
CCK	caspase cleaved cytokeratin (8 & 18)- M30-M 65
C_2_H_5_OH	ethyl alcohol, ethanol
CoA	coenzyme A
COL1A1	collagen 1A1
CPT-1	carnitin-palmitoyl-transferase1
CXCL	chemokine
CYP2E1	cytochrome P4502E1
DAMPs	danger-associated molecular patterns
DNA	deoxyribonucleic acid
EGF	epithelial growth factor
Egr-1	early growth response 1
FA	fatty acids
FABP	fatty acid binding protein
FASN	fatty acid synthetase
FDA	Food and Drug Administration
FFA	free fatty acid
FL	fatty liver
FN1	fibronectin 1
GGT	γ-glutamyl-transferase
GSH	glutathione
H3K9	tri-methylation of lysine 9 of histone 3
HER	hydroxyethyl radical
HGF	hepatocyte growth factor
ICD	International Classification of Diseases
IFN	interferon
IL	interleukine
LPB	LPS binding protein
LPS	lipopolysaccharides
KC	Kupfer cells
MAA	malondialdehyde
MAFLD	metabolic associated fatty liver disease
MEOS	microsomal ethanol oxidizing system
Mean ± SD	mean ± standard deviation
mTOR	mammalian target of rapamycin
NAD^+^	nicotin-amide-adenine dinucleotide
NADH	nicotin-amide-adenine dinucleotide-hydrogen
NADP^+^	nicotinamide adenine dinucleotide phosphate
NADPH	reduced nicotinamide adenine dinucleotide phosphate
NRF2	nuclear factor-erythroid 2-related factor 2
PAMP	pathogen-associated molecular patterns
PhET	phosphatidyl ethanol
PPAR alpha	peroxisome proliferator—activated receptor alpha
PRRs	pattern-recognition receptors
PUFA	poly-unsaturated fatty acids
ROS	reactive oxygen specie
SIRT1	sirtuin 1
SRPK2	serine-arginine-rich protein kinase 2 (SRPK2)
SOCS	suppressor of cytokine signalling
SRABP1c	sterol element binding protein 1c
SREBP-1	sterol-regulatory element binding protein-1
STAT3	signal transducers and activator of transcriptor 3
TGF-β	transforming growth factor-β
TIMP1	TIMP metallopeptidase inhibitor
TLR4	toll-like receptor 4
TNF α	tumour necrosis factor α
VLDL	very-low-density lipoproteins
